# 18‑α‑glycyrrhetinic acid induces apoptosis in gingival fibroblasts exposed to phenytoin

**DOI:** 10.3892/etm.2024.12586

**Published:** 2024-05-22

**Authors:** Reiri Takeuchi, Takatoshi Nomura, Manabu Yaguchi, Chieko Taguchi, Itaru Suzuki, Haruka Suzuki, Hiroko Matsumoto, Yuichiro Okada, Kazumune Arikawa, Takato Nomoto, Koichi Hiratsuka

**Affiliations:** 1 Department of Biochemistry and Molecular Biology, Nihon University School of Dentistry at Matsudo, Matsudo, Chiba 271-8587, Japan; 2 Department of Special Needs Dentistry, Nihon University School of Dentistry at Matsudo, Matsudo, Chiba 271-8587, Japan; 3 Department of Special Needs Dentistry, Nihon University Graduate School of Dentistry at Matsudo, Matsudo, Chiba 271-8587, Japan; 4 Department of Preventive and Public Oral Health, Nihon University School of Dentistry at Matsudo, Matsudo, Chiba 271-8587, Japan; 5 Department of Pharmacology, Nihon University School of Dentistry at Matsudo, Matsudo, Chiba 271-8587, Japan; 6 Department of Histology, Nihon University School of Dentistry at Matsudo, Matsudo, Chiba 271-8587, Japan

**Keywords:** gingival overgrowth, PHT, 18α-GA, gingival fibroblast, apoptosis, death receptor pathway

## Abstract

Phenytoin (PHT)-induced gingival overgrowth is caused by the increased proliferation and reduced apoptosis of gingival fibroblasts in inflammatory gingiva. Licorice has long been used as a component of therapeutic preparations. It inhibits cell proliferation, induces cell apoptosis and has anti-inflammatory effects. 18-α-glycyrrhetinic acid (18α-GA), the active compound in licorice, promotes apoptosis in various types of cells. The present study determined whether 18α-GA affects apoptosis in gingival fibroblasts exposed to PHT. The present study aimed to establish a basis for the therapeutic application of 18α-GA to treat the gingival overgrowth induced by PHT. Human gingival ﬁbroblasts from healthy donors were cultured to semi-conﬂuence and then stimulated in serum-free DMEM containing PHT with or without 18α-GA for subsequent experiments. Apoptotic cells were detected by ELISA. Analysis of the distribution of cell cycle phases and the apoptotic cell population was performed by flow cytometry. The expression levels of mRNAs and proteins of apoptotic regulators were measured using reverse transcription-quantitative PCR and western blotting, respectively. Caspase (CASP) activities were assessed by an ELISA. Treatment with 18α-GA markedly increased the number of apoptotic cells, reduced BCL2 mRNA expression, increased CASP2 and receptor (TNFRSF)-interacting serine-threonine kinase 1 (RIPK1) domain containing adaptor with death domain, Fas (TNFRSF6)-associated via death domain, RIPK1, tumor necrosis factor receptor superfamily; member 1A, TNF receptor-associated factor 2, CASP2, CASP3 and CASP9 mRNA expression, and also upregulated the protein expression levels and activities of caspase-2, caspase-3 and caspase-9. These results demonstrated that 18α-GA induced apoptosis through the activation of the Fas and TNF pathways in the death receptor signaling pathway in gingival fibroblasts treated with PHT. 18α-GA exhibited therapeutic potential for the treatment of PHT-induced gingival overgrowth.

## Introduction

Phenytoin (PHT, an antiepileptic drug), cyclosporin A (an immunosuppressant) and nifedipine (a calcium channel blocker) cause gingival overgrowth as side effects (1-3). Among those drugs, a high probability of gingival overgrowth caused by PHT is known (approximately 50%) (4). A characteristic of gingival overgrowth in clinical conditions is an increase in the size of the gingiva (5). Overgrowth of the gingiva not only disrupts normal mastication but also results in unusual facial features that can cause mental distress (3,6-8). Basic periodontal treatments and surgery are usually performed to improve gingival overgrowth (9), however, an effective medication has not been identified at the present time.

Based on histological observations, PHT-induced gingival overgrowth has characteristic increases in the proliferation of fibroblasts and accumulated amounts of collagen in the gingiva (3,10,11). Gingival fibroblasts are the primary cell type in gingival connective tissue and their role is the maintenance and repair of that tissue (12). The pathogenic mechanisms responsible for PHT-associated gingival overgrowth have been determined using an *in vitro* model and the effects of PHT on gingival fibroblasts in tissue culture have been investigated (13-17). Fibroblast proliferation is observed in periodontal tissues with PHT-induced gingival overgrowth (18). Furthermore, the interaction of drugs with inflammation causes the increased growth and reduced apoptosis of gingival fibroblasts, and consequently the overgrowth of gingiva proceeds (18,19).

Licorice has long been used as a medicinal herb and as a sweetener to give sweetness to food products (20,21). It contains many phytochemicals including more than 300 flavonoids and 20 triterpenoids (22), and it also inhibits mild inflammation and heals ulcers. In addition, licorice inhibits cell proliferation through blocking the cell cycle in mammalian cells (23), and it also induces apoptosis (24). 18-alpha-Glycyrrhetinic acid (18α-GA) is a bioactive compound extracted from licorice that exhibits many biological and pharmacological effects such as anti-inflammatory effects (25). 18α-GA is also apoptotic promoter in epithelial cell rests of Malassez (24). Also, 18α-GA induces apoptosis in leukemic HL60 cells (26) and in ovarian cancer A2780 cells (27). These ﬁndings suggest that 18α-GA could be used to treat PHT-influenced gingival overgrowth since it may induce the apoptosis of gingival ﬁbroblasts.

In this research, we investigated the effects of 18α-GA on apoptosis and on apoptotic regulators in gingival ﬁbroblasts exposed to PHT, to evaluate the therapeutic potential of 18α-GA. The results show that 18α-GA regulates caspase activity in the death receptor pathway in gingival fibroblasts, which results in the induction of apoptosis.

## Materials and methods

### Cell culture

**Figure 1:**
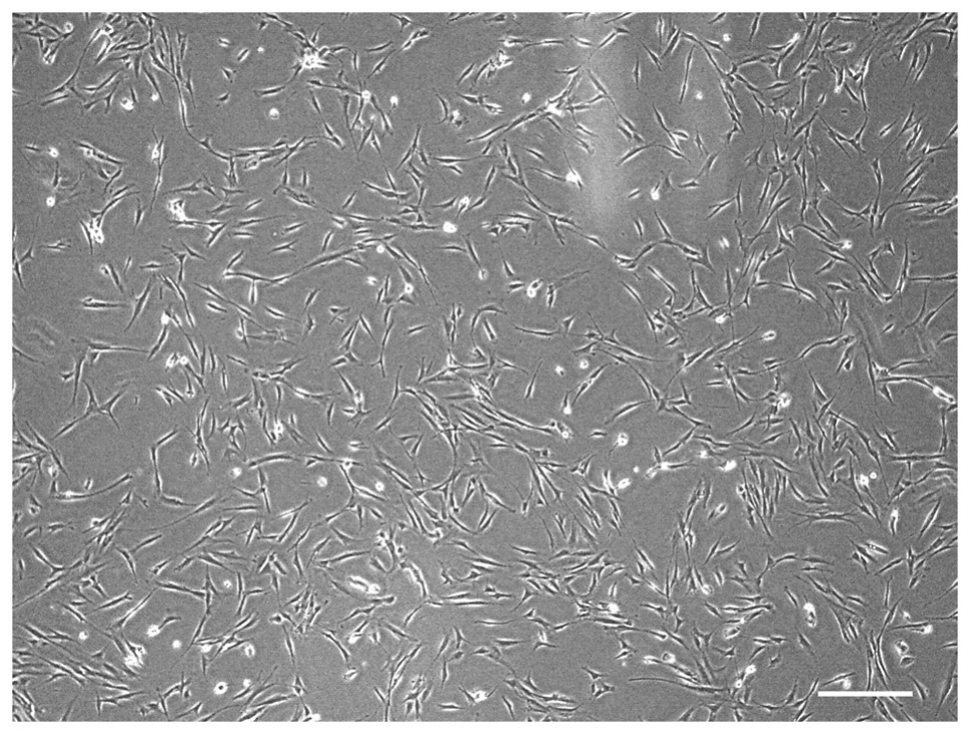
A representative cell image from four independent experiments is shown. Cells were cultured in an atmosphere of 5% CO_2_/95% air at 37˚C in D-MEM (high glucose) with L-glutamine and Phenol Red supplemented with 10% FBS, 50 units/ml penicillin and 50 µg/ml streptomycin to reach semi-conﬂuence. Cells were routinely passaged using 0.05 w/v% trypsin-0.53 mmol/l EDTAx4Na Solution with Phenol Red. Scale bar, 100 µm.

The methods used in this study are based on previously published reports (3,17,20,28,29). PHT and 18α-GA were purchased from Sigma-Aldrich, Japan K.K. (Tokyo, Japan). Four primary cultures of ﬁbroblasts derived from the gingiva of healthy donors were obtained from ScienCell™ Research Laboratories (cat. no. 2620, https://sciencellonline.com/human-gingival-fibroblasts/, San Diego, CA, USA). Those cells had been cryopreserved at passage one and delivered frozen. Cells were cultured in an atmosphere of 5% CO_2_/95% air maintained at 37˚C in Dulbecco's modified Eagle medium (High Glucose) with L-Glutamine and Phenol Red (D-MEM, FUJIFILM Wako Pure Chemical Corporation, Osaka, Japan) supplemented with 10% foetal bovine serum, 50 units/ml penicillin and 50 µg/ml streptomycin (Gibco, Thermo Fisher Scientific, Inc., Waltham, MA, USA) until they reached semi-conﬂuence. Cells were routinely passaged using 0.05 w/v% Trypsin-0.53 mmol/l EDTA·4Na Solution with Phenol Red (FUJIFILM Wako Pure Chemical Corporation). Cells were used between passages 6 and 9 for subsequent experiments (Fig. 1). The concentrations of PHT and 18α-GA used in this study were decided according to the results of previous studies as follows: 0.25 µM PHT significantly inhibited the G_1_ cell cycle arrest and increased the cell proliferation of gingival fibroblasts compared with the untreated control (17,29); 10 µM 18α-GA significantly decreased the proliferation of gingival fibroblasts compared with 0, 0.1, and 1 µM 18α-GA (20).

### Apoptosis assay

Apoptosis assays were performed using an APOPercentage™ Apoptosis Assay Kit (BiocolourLtd., Northern Ireland, UK). After semiconfluent cells were treated with 0.25 µM PHT with or without 10 µM 18α-GA in serum-free D-MEM for 24, 48 and 72 h, the apoptotic cells were labelled with APOPercentage Dye in fresh D-MEM at 37˚C in 5% CO_2_ for 1 h. The D-MEM containing the dye was removed, after which the APOPercentage Dye release reagent was added into the cell culture plates and the plates were gently shaken for 10 min. The absorbance of the released dye at 550 nm was then determined. The methods used in this study are based on previously published reports (3,17,20).

### Propidium iodide staining and ﬂow cytometric analysis

The propidium iodide staining and ﬂow cytometric analysis were performed using a CycleTEST™ plus DNA Reagent Kit (Becton Dickinson and Company, Franklin Lakes, NJ, USA; BD). After semiconfluent cells were treated with 0.25 µM PHT with or without 10 µM 18α-GA in serum-free D-MEM for 48 h, cells were harvested by trypsinization, washed three times with Buffer Solution, and then treated with Solution A (trypsin buffer), Solution B (trypsin inhibitor and RNase buffer) and Solution C (PI stain solution) in accordance with the manufacturer's instructions. A BD FACSCalibur™ Flow Cytometer (BD Biosciences) acquired 20,000 events for each sample, and the percentage of cells in the Sub-G_1_ (apoptotic), G_0_/G_1_, S and G_2_/M phases of the cell cycle were determined using BD CellQuest Pro Software (version 3.1, BD Biosciences). The methods used in this study are based on previously published reports (3,17,20).

### RNA isolation and reverse transcription-quantitative PCR (RT-qPCR)

After semiconfluent cells were treated with 0.25 µM PHT with or without 10 µM 18α-GA in serum-free D-MEM for 12 h, total RNA was immediately extracted from the cells using a RNeasy Mini Kit (QIAGEN, Tokyo, Japan). A standard spectrophotometric method was used to assess the concentration and purity of each extracted total RNA. One µg of each total RNA was then reverse-transcribed using a PrimeScript™ RT reagent Kit (TAKARA BIO INC., Shiga, Japan). The cDNAs were analyzed by qPCR in an Eco™ Real-Time PCR System (Illumina, Inc., San Diego, CA, USA) using a KAPA SYBR^®^ FAST qPCR Master Mix Kit (KAPA BIOSYSTEMS Inc., Wilmington, MA, USA). The following thermocycling conditions were used for qPCR: Enzyme activation at 95˚C for 30 sec, followed by 45 cycles of denaturation at 95˚C for 5 sec and annealing and extension at 60˚C for 20 sec. A Perfect Real Time Support System (TAKARA BIO INC.) was used to synthesize the following PCR primers: B-cell CLL/lymphoma 2 (BCL2); baculoviral IAP repeat containing 3 (BIRC3); CASP8 and FADD-like apoptosis regulator (CFLAR); CASP2 and RIPK1 domain containing adaptor with death domain (CRADD); Fas (TNFRSF6)-associated via death domain (FADD); receptor (TNFRSF)-interacting serine-threonine kinase 1 (RIPK1); tumor necrosis factor receptor superfamily; member 1A (TNFRSF1A); TNF receptor-associated factor 2 (TRAF2); and glyceraldehyde-3-phosphate dehydrogenase (GAPDH). These primer sequences were used according to the method of Takeuchi *et al* (3). The PCR primers for Caspases-2, -3, -8, -9 and -10 were synthesized by Custom DNA Oligos (Merck KGaA, Darmstadt, Germany) and Primer-BLAST (National Library of Medicine, Bethesda, MD, USA). Primer sequences used are listed in Table I. Relative quantiﬁcation was calculated using the 2^-∆∆Cq^ method (30). After normalization to GAPDH, RNA ratios in treated vs. control cultures were determined. The methods used in this study are based on previously published reports (3).

### Western blot analysis

After semiconfluent cells were treated with 0.25 µM PHT with or without 10 µM 18α-GA in serum-free D-MEM for 24 h, the cells were washed with 37˚C PBS and lysed using β-ME Sample Treatment for Tris SDS (COSMO BIO Co., LTD, Tokyo, Japan). Protein concentrations were determined using a TaKaRa Bradford Protein Assay Kit (TAKARA BIO INC.). Equal quantities of protein extracts (10 µg/lane) were separated via SDS-PAGE with Running Buffer Solution for SDS-PAGE (NACALAI TESQUE, INC., Kyoto, Japan) after which the proteins were transferred to PVDF membranes. The membranes were blocked for 30 min at room temperature with Bullet Blocking One for Western Blotting (NACALAI TESQUE), after which they were probed at room temperature for 1 h with primary antibodies against Caspase-2 (1:500), Caspase-3 (1:1,000), Caspase-9 (1:1,000) and β-Actin (1:1,000). The membranes were washed three times with Tris Buffered Saline with 0.05%-Detergent (NACALAI TESQUE) for 5 min at room temperature, and were then incubated with the secondary antibody (1:10,000) at room temperature for 45 min. The primary and secondary antibodies were diluted using Can Get Signal^®^ Immunoreaction Enhancer Solution (TOYOBO CO., LTD., Osaka, Japan). After washing, the blots were detected using Chemi-Lumi One Super (NACALAI TESQUE) and ChemiDoc™ MP Imaging System (Bio-Rad Laboratories, Inc., Hercules, CA, USA). The densities of western blot bands were measured using ImageJ (1.53t; Java 1.8.0_345 [64-bit]). Primary antibodies against Caspase-3 (cat. no. #9662), Caspase-9 (cat. no. #9502) and β-Actin (cat. no. #4967), as well as anti-rabbit HRP-conjugated IgG were purchased from Cell Signaling Technology, Inc. (Danvers, MA, USA). Rabbit monoclonal anti-Caspase-2 antibody (cat. no. ab32021) was purchased from Abcam plc. (Cambridge, UK). The secondary antibody (anti-rabbit IgG, HRP-linked antibody; cat. no. #7074) was purchased from Cell Signaling Technology. The methods used in this study are based on previously published reports (3).

### Detection of caspase activity

After semiconfluent cells were treated with 0.25 µM PHT with or without 10 µM 18α-GA in serum-free D-MEM for 24 h, Caspase-2, -3 and -9 Colorimetric Assay Kits (Medical & Biological Laboratories Co., Ltd., Nagoya, Japan) and a spectrophotometer at 405 nm were used according to the manufacturer's protocols to assess caspase activities. Caspase-2, caspase-3 and caspase-9 were labelled using the synthetic peptide substrates VDVAD-*p*-nitroanilide (*p*NA), DEVD-*p*NA and LEHD-*p*NA respectively. The methods used in this study are based on previously published reports (3,20,28).

### Statistical analysis

All data are reported as mean ± standard error of the mean (SEM). Statistical analysis was carried out using Welch's t-test. *P* values <0.05 were considered to indicate a statistically signiﬁcant difference.

## Results

### Relative number of apoptotic cells after treatment of gingival fibroblasts with 18α-GA

**Figure 2:**
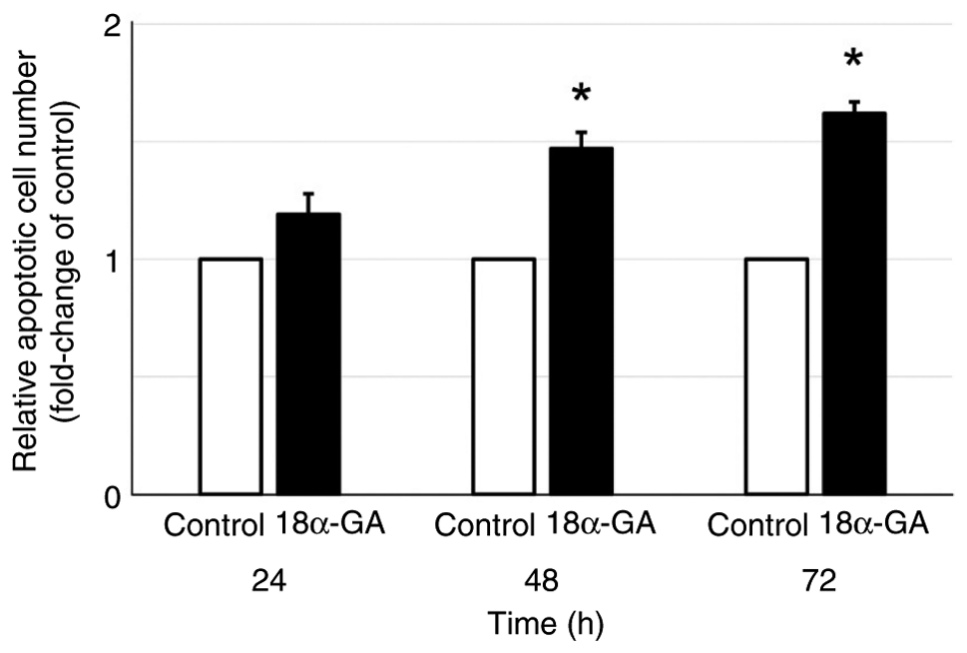
Relative apoptotic cell number in gingival fibroblasts treated with PHT in the presence or absence of 18α-GA. After semiconfluent cells were treated with 0.25 µM PHT with or without (control) 10 µM 18α-GA in serum-free D-MEM for 24, 48 and 72 h, the quantification of apoptotic cells was performed by detecting the absorbance at 550 nm using APOPercentage Dye. After normalization to 0 h, the fold change compared with the control was determined. Data are presented as the mean ± SEM. ^*^P<0.05 compared with the control using Welch's t-test (n=4). 18α-GA, 18-α-glycyrrhetinic acid; PHT, phenytoin.

Apoptosis was assessed in gingival fibroblasts after treatment with or without 18α-GA. As shown in Fig. 2, gingival fibroblasts treated with 18α-GA showed a time-dependent increase in the relative number of apoptotic cells compared to the untreated control with significant increases at 48 h (1.5-fold) and at 72 h (1.6-fold).

### Apoptotic population and cell cycle dynamics of gingival fibroblasts treated with 18α-GA

**Figure 3:**
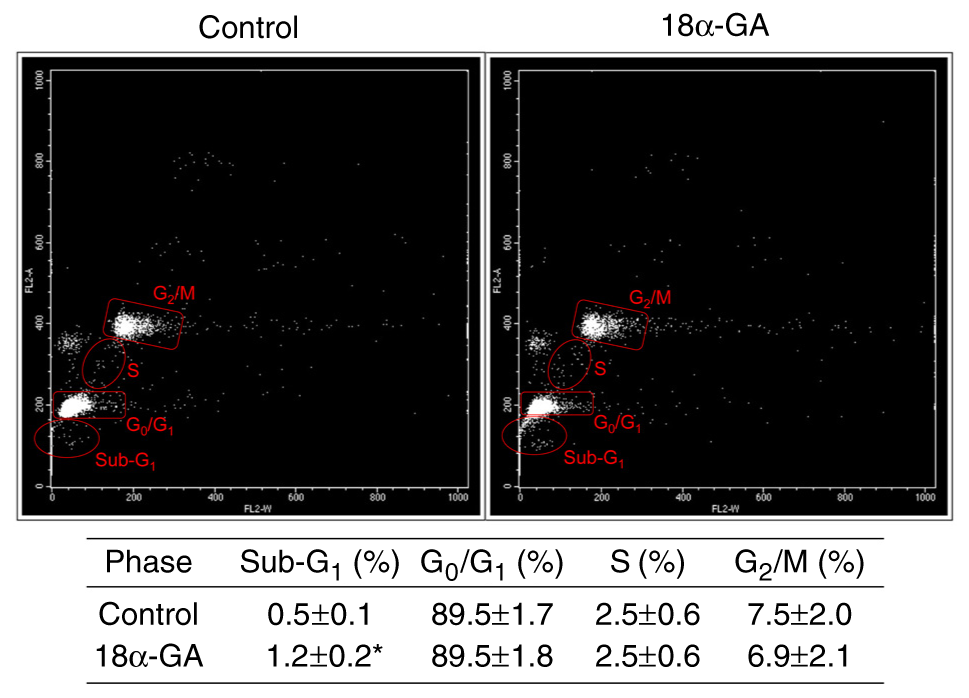
Analysis of the apoptotic cell population (sub-G_1_) and distribution of cell cycle phases of gingival fibroblasts cultured in the presence or absence of 18α-GA. Semiconfluent cells were incubated in serum-free D-MEM containing phenytoin (0.25 µM) with or without (control) 18α-GA (10 µM) for 48 h and then subjected to flow cytometric analysis. A representative dot plot from four independent experiments is shown. The detailed values of sub-G_1_ and cell cycle parameters are shown in the table at the bottom. The data are presented as the mean ± SEM. ^*^P<0.05 compared with the control using Welch's t-test (n=4). 18α-GA, 18-α-glycyrrhetinic acid; FL2-A, fluorescence pulse signal 2-area; FL2-W, fluorescence pulse signal 2-width.

We analyzed the apoptotic cell population (sub-G_1_) and the distribution of cell cycle phases (G_0_/G_1_, S, and G_2_/M) in gingival fibroblasts treated with or without 18α-GA. As shown in Fig. 3, treatment with 18α-GA significantly increased the number of apoptotic cells, however it did not change the distribution of cells in the G_0_/G_1_, S and G_2_/M phase.

### mRNA expression levels in gingival fibroblasts treated with 18α-GA

**Figure 4:**
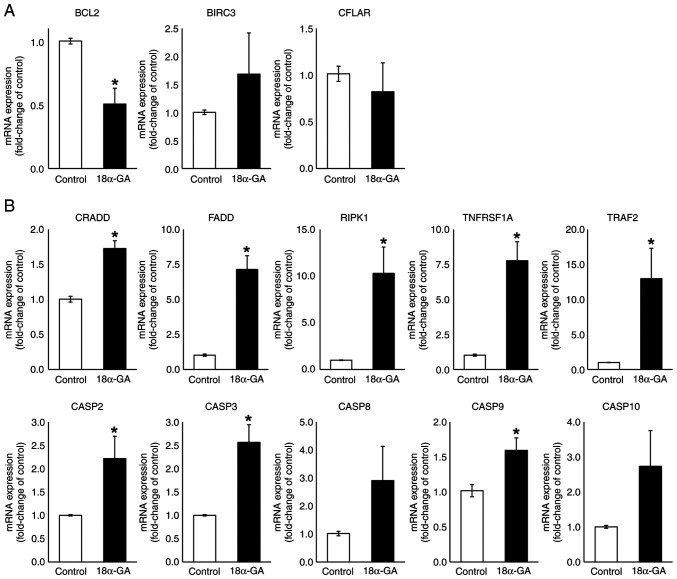
mRNA expression levels of apoptotic regulators in gingival fibroblasts treated with PHT in the presence or absence of 18α-GA. Semiconfluent cells were incubated in serum-free D-MEM containing PHT (0.25 µM) with or without (control) 18α-GA (10 µM) for 12 h, after which reverse transcription-quantitative PCR analysis was performed. Relative quantification was performed using the 2^-∆∆Cq^ method. After normalization to GAPDH, RNA ratios in treated vs. control cultures were determined. Data are presented as the mean ± SEM. ^*^P<0.05 compared with the control using Welch's t-test (n=4). (A) Anti-apoptotic genes. (B) Pro-apoptotic genes. 18α-GA, 18-α-glycyrrhetinic acid; BIRC3, baculoviral IAP repeat containing 3; CASP, caspase; CFLAR, CASP8 and FADD-like apoptosis regulator; CRADD, CASP2 and RIPK1 domain containing adaptor with death domain; FADD, Fas (TNFRSF6)-associated via death domain; PHT, phenytoin; RIPK1, receptor (TNFRSF)-interacting serine-threonine kinase 1; TNFRSF1A, tumor necrosis factor receptor superfamily; member 1A; TRAF2, TNF receptor-associated factor 2.

We analyzed the effects of 18α-GA treatment of gingival fibroblasts on their mRNA expression levels of apoptotic factors (BCL2, BIRC3, CFLAR, CRADD, FADD, RIPK1, TNFRSF1A, TRAF2, CASP2, CASP3, CASP8, CASP9 and CASP10) using qPCR. As shown in Fig. 4, the treatment of gingival fibroblasts with 18α-GA significantly reduced the BCL2 (0.5-fold) mRNA expression level and significantly increased CRADD (1.7-fold), FADD (7.1-fold), RIPK1 (10.3-fold), TNFRSF1A (7.8-fold) and TRAF2 (13.0-fold) mRNA expression levels. Treatment with 18α-GA also increased the BIRC3 (1.7-fold) mRNA expression level and decreased the CFLAR (0.8-fold) mRNA expression level but not significantly. Treatment of gingival fibroblasts with 18α-GA also significantly increased CASP2 (2.2-fold), CASP3 (2.6-fold) and CASP9 (1.6-fold) mRNA expression levels and increased CASP8 (2.9-fold) and CASP10 (2.7-fold) mRNA expression levels but not significantly.

### Protein expression in gingival fibroblasts treated with 18α-GA

**Figure 5:**
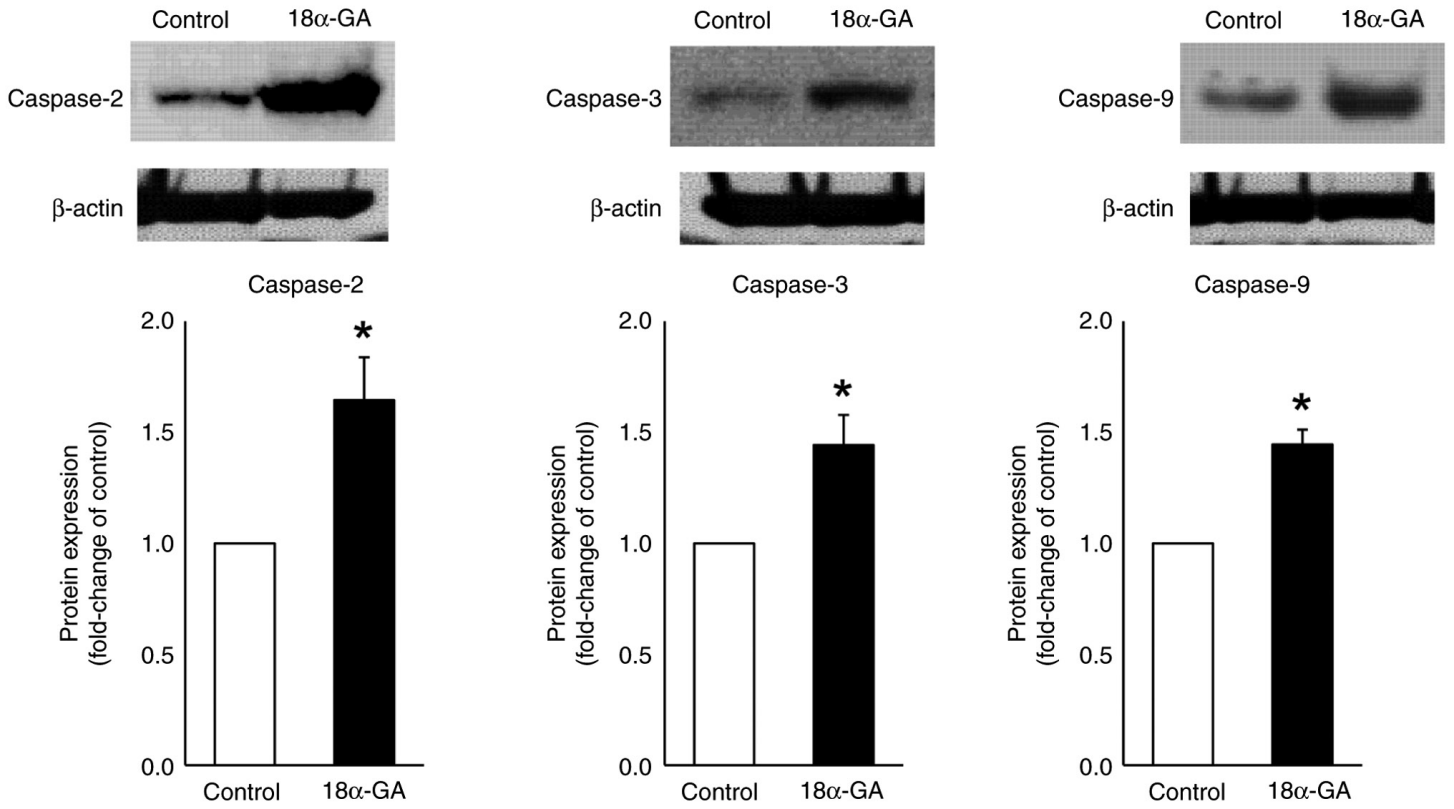
Protein expression levels of caspases in gingival fibroblasts treated with PHT in the presence or absence of 18α-GA. Semiconfluent cells were incubated in serum-free D-MEM containing PHT (0.25 µM) with or without (control) 18α-GA (10 µM) for 24 h and then assessed using western blotting, after which the fold change compared with the control was determined. The band images shown are representative of results from four independent experiments. Data are presented as the mean ± SEM. ^*^P<0.05 compared with the control using Welch's t-test (n=4). 18α-GA, 18-α-glycyrrhetinic acid; PHT, phenytoin.

We analyzed the effects of 18α-GA treatment of gingival fibroblasts on the protein expression of caspases- 2, 3 and 9 using western blot analysis. As shown in Fig. 5, treatment of gingival fibroblasts with 18α-GA significantly increased the protein expression levels of caspase-2 (2.3-fold), caspase-3 (2.7-fold) and caspase-9 (2.8-fold) compared to the levels observed in control cells.

### Caspase activity in gingival fibroblasts treated with 18α-GA

**Figure 6:**
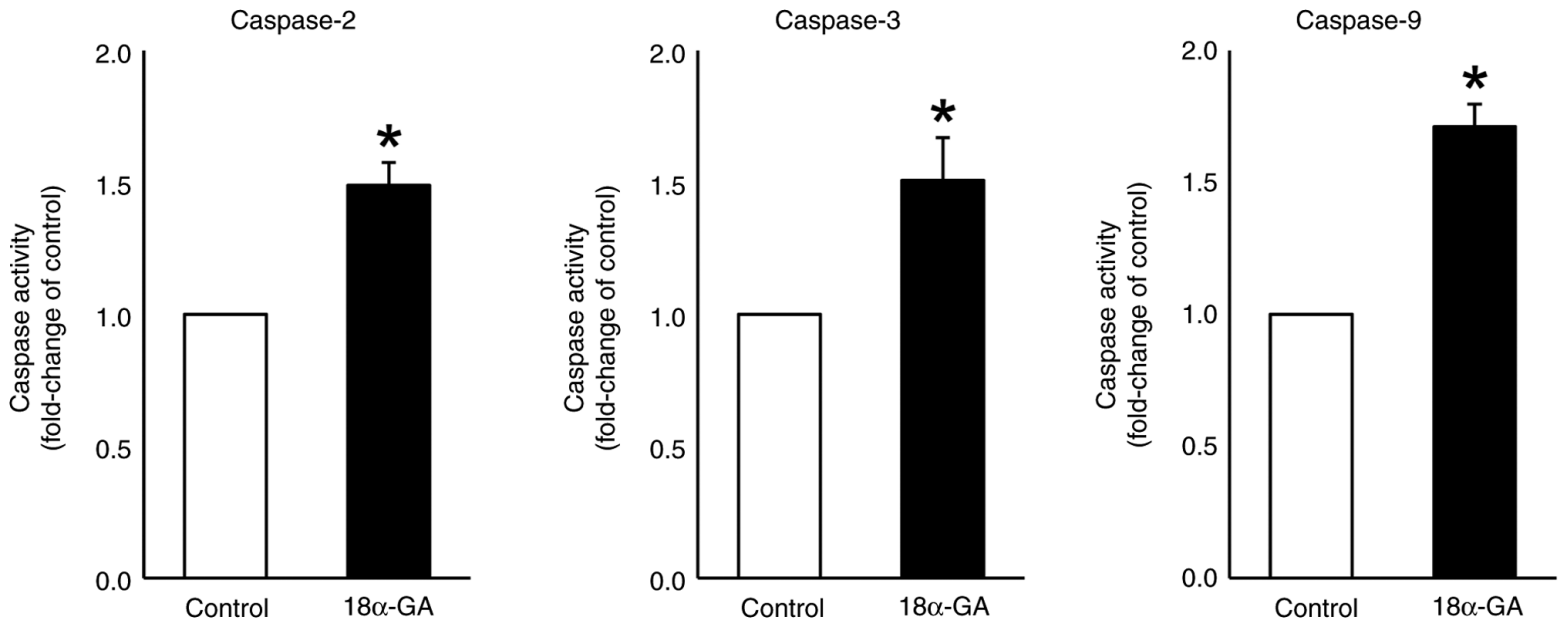
Caspase activity in gingival fibroblasts treated with PHT in the presence or absence of 18α-GA. After semiconfluent cells were treated with 0.25 µM PHT with or without (control) 10 µM 18α-GA in serum-free D-MEM for 24 h, caspase activities were assessed by detecting the absorbance at 405 nm, after which the fold change compared with the control was determined. Data are presented as the mean ± SEM. ^*^P<0.05 compared with the control using Welch's t-test (n=4). 18α-GA, 18-α-glycyrrhetinic acid; PHT, phenytoin.

We assessed the effects of treating gingival fibroblasts with 18α-GA on the activities of caspases- 2, 3 and 9. As shown in Fig. 6, treatment with 18α-GA significantly up-regulated the activities of caspase-2 (1.5-fold), caspase-3 (1.5-fold) and caspase-9 (1.7-fold) compared to the levels observed in control cells.

## Discussion

In the present study, we determined whether treatment with 18α-GA affects apoptosis of gingival fibroblasts exposed to PHT. The purpose of this study was to establish a basis for the therapeutic application of 18α-GA to treat PHT-induced gingival overgrowth. We found that 18α-GA induced the apoptosis of gingival fibroblasts by activating the caspase cascade in the death receptor pathway.

Gingival overgrowth is caused by the increased proliferation and the decreased apoptosis of gingival fibroblasts that are exposed to drugs, such as PHT (3). The pathogenesis of this disease is also associated with the gingiva including inflammation (31). 18α-GA promotes the apoptosis of multiple types of cells, including porcine epithelial cell rests of Malassez cells (24), leukemic HL60 cells (26), ovarian cancer A2780 cells (27) and murine microglial BV2 cells (32). In this study, we found that 18α-GA induced the apoptosis of gingival fibroblasts. Conversely, transformed cells with severe DNA damage are cleared by cellular apoptosis (33). Several studies have proposed that the pathogenesis of gingival overgrowth involves the inhibition of apoptosis (3,34,35). We have also demonstrated that gingival overgrowth is attributed to reduced apoptosis in gingival ﬁbroblasts derived from patients with gingival overgrowth (28).

Apoptosis is programmed cell death characterized by an elaborate sequence of morphological events including nuclear condensation (pyknosis) and fragmentation (karyorrhexis), along with blebbing of the plasma membrane, both of which contribute to the formation of apoptotic bodies (36,37). The two main pathways of apoptotic cell death are the intrinsic and extrinsic pathways. The intrinsic pathway is marked by mitochondrial outer membrane permeabilization, which releases cytochrome c from the mitochondrial intermembrane space (38). The extrinsic pathway is activated in response to specific death receptors which are Fas, TNF receptor 1 (TNFR1) or TNF-related apoptosis-inducing ligand receptor. Both pathways trigger downstream effector caspases such as caspase-3 that lead to apoptotic cell death (36,39). The expression of caspase-3 protein is attenuated in tissues of gingiva derived from patients treated with cyclosporin A and/or nifedipine, and from those with PHT-induced gingival overgrowth (3,18,35). PHT treatment of gingival fibroblasts from healthy donors also reduced the expression and activity of caspase-3(3). In this study, we show that treatment with 18α-GA enhances the mRNA and protein expression levels of caspase-3 and increases the activation of caspase-3.

To elucidate the pro-apoptotic mechanism of 18α-GA in the death receptor pathway of gingival fibroblasts exposed to PHT, we examined the mRNA expression levels of apoptotic genes, including inducers (CRADD, FADD, RIPK1, TNFRSF1A and TRAF2), an effector (CASP3), initiators (CASP2, CASP8, CASP9 and CASP10), inhibitors (BCL2, BIRC3 and CFLAR) and the protein expression levels and activities of caspases (2, 3 and 9) in those cells. We found that treatment with 18α-GA reduced the mRNA expression level of BCL2, enhanced the mRNA expression levels of CASP2, CASP3, CASP9, CRADD, FADD, RIPK1, TNFRSF1A and TRAF2, and increased the protein expression levels and activities of caspase-2, caspase-3 and caspase-9 in gingival fibroblasts treated with PHT.

As mentioned above, the major death receptors are Fas and TNFR1. After those receptors are activated by extracellular ligands such as Fas ligand (Fas-L) and TNF ligands, initiator caspases (2, 8, 9 and 10) activate executioner caspases (3, 6 and 7) in cooperation with various apoptotic mediators, and consequently, apoptosis is induced (40). When Fas is bound by its ligand Fas-L, the resulting conformational change in Fas DD (Fas death domain) allows it to bind to FADD DD. The binding of Fas DD to FADD DD causes the exposed DED (death effector domain) on FADD (40). The binding of TNF-α (one of the TNF ligands) to TNF receptor-1, which is encoded by TNFRSF1A, results in the recruitment of the adapter proteins FADD and TRADD (3,41-43). FADD then activates caspase-8 and caspase-10. Caspase-8 can cleave and activate the apoptosis executioner caspase-3(40). The action of c-FLIP, which is encoded by CFLAR, prevents FADD recruitment (44,45). On the other hand, when the adapter protein TRAF2 binds to the TNF receptor-1/TRADD complex, NF-κB is activated and apoptosis is inhibited (46). c-IAP2, which is encoded by BIRC3, inhibits the activation of NF-κB (47,48). A complex consisting of CRADD-activated caspase-2, RIPK1 and TNF receptor-1 activates caspase-9 and caspase-3, which can induce apoptosis (49). BCL2 prevents apoptosis by depressing the activation of caspase-9(50).

**Figure 7:**
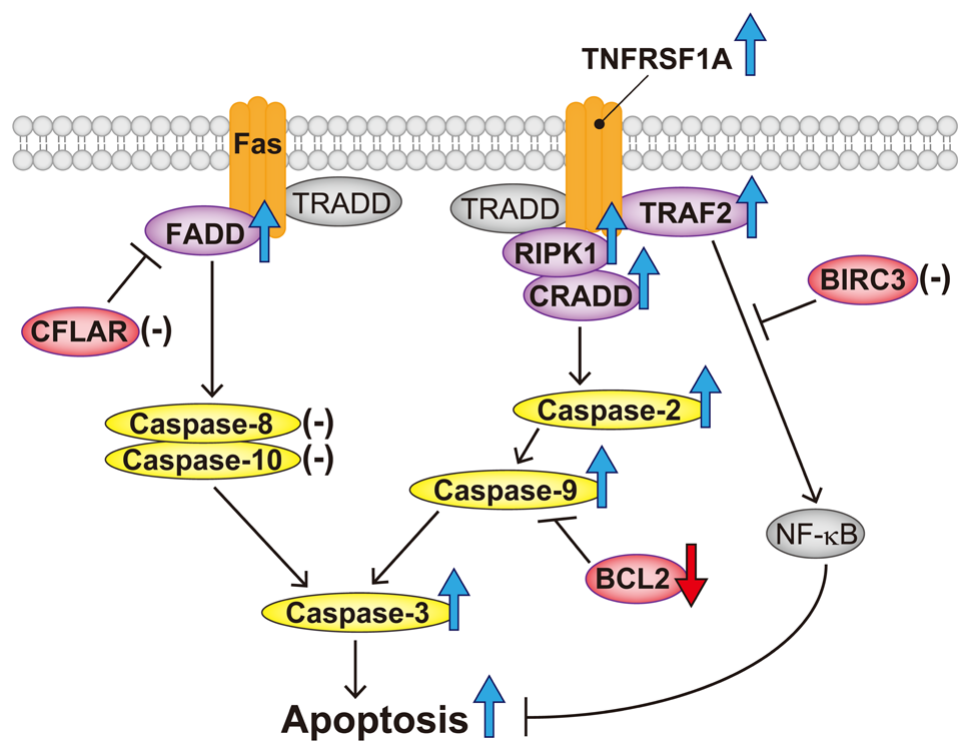
Schematic representation of apoptosis accelerated by 18α-GA in gingival ﬁbroblasts treated with phenytoin. 18α-GA induced the upregulation of FADD and caspase-3, leading to an increase in the apoptotic Fas pathway. 18α-GA also induced the upregulation of RIPK1, CRADD, caspase-2, caspase-9 and caspase-3 in the TNF pathway, which resulted in apoptosis acceleration. Furthermore, 18α-GA decreased BCL2, which increased caspase-9. Purple (components of death-inducing signaling complex), red (antiapoptotic factors) and yellow (caspases) ellipses denote the molecules analyzed in the present study. The blue or red large arrows denote upregulation or downregulation, respectively, following 18α-GA treatment. Hyphens denote the molecules that are unaffected by 18α-GA treatment. 18α-GA, 18-α-glycyrrhetinic acid; BIRC3, baculoviral IAP repeat containing 3; CFLAR, CASP8 and FADD-like apoptosis regulator; CRADD, CASP2 and RIPK1 domain containing adaptor with death domain; FADD, Fas (TNFRSF6)-associated via death domain; RIPK1, receptor (TNFRSF)-interacting serine-threonine kinase 1; TNFRSF1A, tumor necrosis factor receptor superfamily; member 1A; TRADD, TNFRSF1A associated via death domain; TRAF2, TNF receptor-associated factor 2.

Our results show that 18α-GA has the following effects on gingival fibroblasts exposed to PHT: a decrease in the BCL2 mRNA expression level; increases in CRADD, FADD, RIPK1, TNFRSF1A and TRAF2 mRNA expression levels; increases in CASP2, CASP3 and CASP9 mRNA expression levels and increases in caspase-2, caspase-3 and caspase-9 protein expression levels and activities. Based on the above results, the apoptotic mechanism of 18α-GA in gingival fibroblasts treated with PHT may be as follows: 18α-GA modulates the TNF pathway by upregulating TNFRSF1A, TRAF2, RIPK1 and CRADD, which induce apoptosis via the activation of caspase-2, caspase-9 and caspase-3; 18α-GA affects the Fas pathway by upregulating FADD and induces apoptosis by downregulating BCL2 (shown schematically in Fig. 7). The release of cytochrome c to the cytoplasm from mitochondria also activates caspase-9(3). Thus, 18α-GA may affect apoptosis via the mitochondrial pathway in gingival fibroblasts.

This study demonstrates that 18α-GA induces apoptosis through activating the pathways of Fas and TNF in the death receptor signaling pathway of gingival fibroblasts treated with PHT. In conclusion, 18α-GA has a therapeutic potential for the treatment of PHT-induced gingival overgrowth. Future studies should investigate the alterations of the mitochondrial pathway in gingival fibroblasts caused by 18α-GA treatment. The mechanism of gingival overgrowth induced by PHT is related to the accumulation of collagen by its enhanced production in numerous gingival fibroblasts (3) or by the impaired metabolism caused by TNF-α and PHT together (7). The fact that TNF-α activates NF-κB may also be related to the accumulation of collagen in the gingiva (3). Thus, future studies should aim to clarify whether 18α-GA affects collagen production/metabolism in gingival fibroblasts exposed to PHT.

## Figures and Tables

**Table I:** Primers used for reverse transcription-quantitative PCR.

Gene symbol	Sequence (5'-3')	Product size, bp	GenBank accession no.
BCL2	F: TGGACAACCATGACCTTGGAC	170	NM_000633.2
	R: GTGCTCAGCTTGGTATGCAGAA		
BIRC3	F: GGACAGGAGTTCATCCGTCAAG	176	NM_001165.3
	R: GCAGCATTAATCACAGGAGTATTCA		
CFLAR	F: TGCAGTTCAGTCAAACATTGGAAG	177	NM_003879.5
	R: GGGTTCCAGATGGTCCAGAAATA		
CRADD	F: CCTAACAGTCAGGATTCCGGTTG	107	NM_003805.3
	R: CGAAGTGAGCGGAGTACTTGTTTG		
FADD	F: ATGCGCGGGTCCCTTAGTT	84	NM_003824.3
	R: CACTCCGGTGCCTGATTCAC		
RIPK1	F: TTTCAAAGCCCACCTGAAACC	170	NM_003804.3
	R: GCCAGATTGACCATCACCACA		
TNFRSF1A	F: ACAGAACACCGTGTGCACCT	103	NM_001065.3
	R: GCACAACTTCGTGCACTCC		
TRAF2	F: GATGGAGGCATCCACCTACGA	163	NM_021138.3
	R: GCCGTTCAGGTAGATACGCAGAC		
CASP2	F: CAGCATGTACTCCCACCGTT	197	NM_032982.4
	R: GCCAGCTGGAAGTGTGTTTG		
CASP3	F: CCTTGAAATCCCAGGCCGT	168	NM_001354777.2
	R: TCCAGAGTCCATTGATTCGCT		
CASP8	F: CTTTCTGGGCACGTGAGGTT	182	NM_001080124.2
	R: CAGGCTCAGGAACTTGAGGG		
CASP9	F: AGGCCCCATATGATCGAGGA	193	NM_001229.5
	R: TCGACAACTTTGCTGCTTGC		
CASP10	F: TCTTGGAAGCCTTACCGCAG	78	NM_032977.4
	R: GTGCACCATTTGTGGCTCTG		
GAPDH	F: GCACCGTCAAGGCTGAGAAC	138	NM_002046.5
	R: TGGTGAAGACGCCAGTGGA		

BIRC3, baculoviral IAP repeat containing 3; CASP, caspase; CFLAR, CASP8 and FADD-like apoptosis regulator; CRADD, CASP2 and RIPK1 domain containing adaptor with death domain; F, forward; FADD, Fas (TNFRSF6)-associated via death domain; R, reverse; RIPK1, receptor (TNFRSF)-interacting serine-threonine kinase 1; TNFRSF1A, tumour necrosis factor receptor superfamily, member 1A; TRAF2, TNF receptor-associated factor 2.

## Data Availability

The datasets used and/or analyzed during the current study are available from the corresponding author on reasonable request.
